# Middle Cerebral Artery Atherosclerotic Plaques in Recent Small Subcortical Infarction: A Three-Dimensional High-resolution MR Study

**DOI:** 10.1155/2015/540217

**Published:** 2015-10-11

**Authors:** Xiao-Dong Zou, Yiu-Cho Chung, Lei Zhang, Ying Han, Qi Yang, Jianping Jia

**Affiliations:** ^1^Department of Neurology, Xuan Wu Hospital, Capital Medical University, Beijing 100053, China; ^2^Paul C. Lauterbur Research Center for Biomedical Imaging, Shenzhen Institutes of Advanced Technology, Chinese Academy of Sciences, Shenzhen 518055, China; ^3^Beijing Key Laboratory of Geriatric Cognitive Disorders, Beijing 100053, China; ^4^Neurodegenerative Laboratory of Ministry of Education of the People's Republic of China, Beijing 100053, China; ^5^Department of Radiology, Xuan Wu Hospital, Capital Medical University, Beijing 100053, China

## Abstract

*Purpose*. Conventional two-dimensional vessel wall imaging has been used to depict the middle cerebral artery (MCA) wall in patients with recent small subcortical infarctions (RSSIs). However, its clinical use has been limited by restricted spatial coverage, low signal-to-noise ratio (SNR), and long scan time. We used a novel three-dimensional high-resolution MR imaging (3D HR-MRI) technique to investigate the presence, locations, and contrast-enhanced patterns of MCA plaques and their relationship with RSSI. *Methods*. Nineteen consecutive patients with RSSI but no luminal stenosis on MR angiography were prospectively enrolled. 3D HR-MRI was performed using a T1w-SPACE sequence at 3.0 T. The presence, locations, and contrast-enhanced patterns of the MCA plaques on the ipsilateral and contralateral sides to the RSSI were analyzed. *Results*. Eighteen patients successfully completed the study. MCA atherosclerotic plaques occurred more frequently on the ipsilateral than the contralateral side to the RSSI (72.2% versus 33.3%, *P* = 0.044). The occurrence of superiorly located plaques was significantly higher on the ipsilateral than the contralateral side of the MCA (66.7% versus 27.8%; *P* = 0.044). *Conclusions*. Superiorly located plaques are closely associated with RSSI. 3D high-resolution vessel wall imaging may be a potential tool for etiologic assessment of ischemic stroke.

## 1. Introduction

Small subcortical infarction (SSI) is a unique entity with a distinct pathogenesis [1]. Traditionally, SSI is believed to be caused by intrinsic diseases of the perforating arterioles [2, 3]. However, the study by Adachi et al. revealed that large artery disease may also lead to SSIs [4]. Distinguishing between the two vasculopathies may help guide optimal therapy. Many conventional modalities exist for imaging luminal stenosis, including CT angiography and MR angiography (MRA). However, they cannot identify atherosclerotic plaques undergoing expansive remodeling on the intracranial vessel wall which do not lead to luminal stenosis. Thus, direct imaging of the intracranial vessel wall itself offers the potential to discriminate such pathologies.

Using two-dimensional high-resolution MR imaging (2D HR-MRI), several studies have demonstrated that atherosclerotic plaques can be detected in patients with single subcortical infarctions (42–60%) [5–8]. Xu et al. compared the characteristics of plaques on the ipsilateral side with those on the contralateral side and reported that superiorly located plaques of middle cerebral artery (MCA) are associated with acute deep brain infarctions [6]. However, 2D technique with relatively low spatial resolution and limited coverage has difficulty identifying small plaques on the vessel walls, especially at the distal tortuous intracranial arteries [9]. Furthermore, there has been limited study of contrast-enhanced high-resolution atherosclerotic plaque imaging in patients with recent SSI (RSSI) but no significant luminal stenosis at the MCA.

In this study, we aimed to compare the presence, locations, and contrast-enhanced patterns of plaques on the ipsilateral and contralateral side of the RSSI using contrast-enhanced three-dimensional HR-MRI (3D HR-MRI).

## 2. Materials and Methods

### 2.1. Study Population

From September 2013 to March 2014, 19 consecutive patients were prospectively recruited into the current study. Patients were enrolled in this study if the following criteria were met: (1) a recent single infarction (maximal infarction diameter ≤ 2 cm) that is believed to be responsible for the clinical symptoms reported within the past 4 weeks: this infarct should have been identified in the territory of the lenticulostriate arteries as confirmed by diffusion-weighted imaging (DWI); (2) no ipsilateral MCA stenosis based on the MRA; (3) one or more risk factors for atherosclerosis (hypertension, diabetes, hyperlipidemia, and smoking). All eligible patients first underwent comprehensive examinations including MRI of the brain (T1- and T2-weighted images, DWI), MRA of the MCA, transcranial Doppler, carotid duplex, electrocardiography, and echocardiography. We excluded patients with the following: (1) one or more stenoses at the ipsilateral intracranial internal carotid artery or relevant extracranial arteries; (2) a definite cardioembolic source; (3) other nonatherosclerotic vasculopathies. Among these patients, those who were unable to undergo a second MRI scan for any reason were also excluded. In this study, the term “recent small subcortical infarction” followed the definition described by Wardlaw et al. [10]. The study protocol was approved by the institutional review board of our hospital. All patients gave written informed consent before undergoing the second MR examination.

### 2.2. Imaging Protocol

The examination was performed on a 3.0 T MRI system (Siemens Magnetom Verio, Erlangen, Germany) using a 32-channel head coil. The examination protocol included 3D time-of-flight MRA, DWI, and pre- and postcontrast 3D T1-weighted HR-MRI.

The 3D HR-MRI was performed using a variant of a 3D turbo spin echo technique known as T1w-SPACE [11]. The technique has good blood suppression properties and high sampling efficiency [12]. The imaging parameters optimized for T1 contrast that were used included the following [13]: TR/TE = 938 ms/24 ms; turbo factor = 29; echo spacing = 4.54 ms; iPAT = 2; average = 1.4 (partial averaging); isotropic voxel resolution varied between (0.5 mm)^3^ and (0.7 mm)^3^, and the average scan time was 8 min (6–10 min, depending on the spatial resolution). For contrast-enhanced T1w-SPACE, 0.1 mmol/kg body weight of gadopentetate dimeglumine (BeiLu Pharmaceutical Co., Ltd, Beijing, China) was manually injected. All subjects included in this study underwent MRI examination within 4 weeks of symptom onset.

### 2.3. Image Analysis

All images were analyzed by two experienced readers. One reader (Qi Yang) is a neuroradiologist with 10-year clinical experience; another reader (Yiu-Cho Chung) is an MR scientist working on plaque imaging for more than 10 years. Both were independently blinded to the patients' clinical information and other images before they reviewed the T1w-SPACE images. The discrepancies between the readers were resolved by consensus reading.

Image analysis was performed on a workstation (Leonardo, Siemens). The infarction at the penetrator territory of the MCA was assessed based on a previously published template [14]. The presence of MCA plaques was identified using both pre- and postcontrast T1w-SPACE images. The two 3D image sets were first coregistered using commercial software (Syngo Fusion, Siemens). In the images, plaques were identified using the criteria by Klein et al. [5]. A plaque has focal wall thickening when it is evident on both the short axis and long axis of the vessel compared with the nearby vessel wall. Care was taken to ensure that the short-axis views of the vessels were perpendicular to the M1 segment of MCA. The end of the M1 segment was defined as the portion at the Sylvian fissure and included the bifurcation [15]. MCAs on both the ipsilateral and contralateral sides of the infarction lesion were reviewed for the presence of plaque.

**Figure 1:**
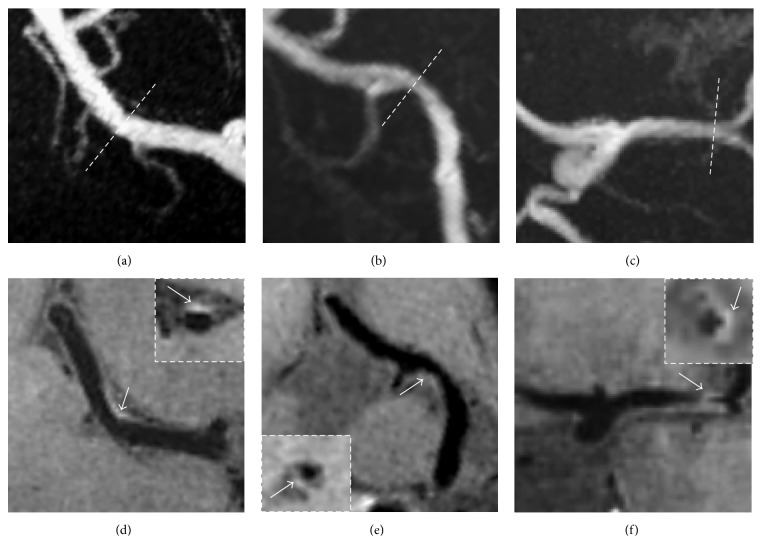
Contrast-enhanced 3D HR-MRI images from three patients. (a)–(c): MRA images show no stenosis on the relevant MCAs in these patients (*short dash line*). (d)–(f): Contrast-enhanced 3D HR-MRI images of the corresponding patients show the plaques and their positions along the MCAs (see arrow): (d) superiorly located superior; (e) inferiorly located plaque; (f) plaque involving both superior and inferior wall.

As most lenticulostriate arteries arise from the superior wall of the MCA trunk [16], the locations of the plaques were analyzed. Based on the position of maximal wall thickening, the cross-sectional locations of the plaques were classified as superior or inferior [8]. A plaque was considered a superiorly located plaque when its maximal thickness was at the superior side of the MCA (Figure 1(d)). Otherwise, it was classified as an inferiorly located plaque (Figure 1(e)). If the plaque thickness was similar on both the superior and inferior sides of the vessel, the plaque was considered to be involved from both sides of the MCA (Figure 1(f)).

Two-dimensional short-axis images of the plaques that were generated from the registered pre- and postcontrast T1w-SPACE images were used to find the plaque enhancement ratio as follows. Region of interest (ROI) of the thickened MCA vessel walls was first defined. The mean signal intensities (SI) of the MCA vessel wall at the ROI on the registered pre- and postcontrast T1w-SPACE were measured. They were then normalized by the SI of the nearby gray matter regions in the pre- and postcontrast images using the method described by Lou et al. [17]. Following [17], a plaque was enhanced when its SI increased by more than 20%.

### 2.4. Statistical Analysis

Continuous data were expressed as mean ± standard deviation. Categorical data were expressed as counts and percentages. The interobserver agreement for the identification of the plaques was assessed by calculating kappa (*κ*). A value of *κ* > 0.8 would indicate almost perfect agreement. Student's *t*-test was used for quantitative data comparison, while Fisher exact test was used for qualitative data comparison. Statistical significance was defined as a *P* value of <0.05. All statistical analyses were performed using SPSS software (SPSS Inc., version 19, Chicago, IL, USA).

## 3. Results

### 3.1. Patients Baseline Characteristics

Eighteen patients successfully completed 3D contrast-enhanced high-resolution vessel wall imaging. One patient was excluded because of suboptimal image quality from the examination. The details of the patients' demographics and main clinical characteristics are shown in Table 1.

### 3.2. Presence and Locations of MCA Plaques

**Figure 2:**
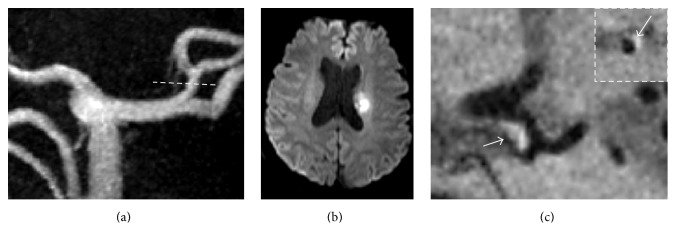
A 56-year-old man with right-side weakness and dysarthria. (a) MRA shows no stenosis on the left MCA; (b) the diffusion-weighted image shows a hypertensive lesion in the left lenticular nucleus with rostral extension to the coronal radiate; (c) the T1-weighted SPACE postcontrast images show apparent eccentric wall thickening (arrow) in close proximity to bifurcation ipsilateral to the infarction on both the long axis and the short axis of the vessel.

Of the 18 patients (totally 36 MCAs), 3D HR-MRI detected 21 plaques that appeared to cause no stenosis on MRA. On precontrast images, 14 plaques were detected and 12 of them were enhanced in postcontrast images. On postcontrast images, MCA atherosclerotic plaques were found in 19 vessels. Superiorly located plaques were found in 17 (89.5%) of the 19 MCAs with plaques. The percentage of atherosclerotic plaques in all the 36 MCAs detected on postcontrast T1w-SPACE images was higher than that on precontrast T1w-SPACE images (52.8% versus 38.9%) (Table 2).  Figure 1 showed three typical cases in which plaques were found at different positions of the MCA arterial wall. The distribution of the plaques found on the ipsilateral and contralateral sides of the lesions was shown in Table 3. Three of the 14 plaques found at the ipsilateral MCAs were located near the bifurcation of the M1 segment.  Figure 2 showed one such case where eccentric vessel wall thickening was located near the bifurcation. MCA plaques occurred more often in the ipsilateral than the contralateral MCA (72.2% versus 33.3%; *P* = 0.044). In addition, the prevalence of superiorly located plaques was significantly higher in the ipsilateral than the contralateral MCA (66.7% versus 27.8%; *P* = 0.044). Weighted *κ* value of the interobserver agreement for the identification of the plaques was 0.828 (95% confidence interval [CI], 0.62–1.00).

### 3.3. Plaque Enhancement

The percentages of superiorly located plaques with increased SI at both the ipsilateral and contralateral MCA were 58.8% ± 34.9% and 72.2% ± 43.5%, respectively (*P* = 0.512 using Student's *t*-test).  Table 4 showed the signal enhancement of the plaques that were centered on the superior side of the MCAs (superiorly located plaques) on both the ipsilateral and contralateral sides of the RSSIs.

## 4. Discussion

In this study, we investigated the MCA atherosclerotic lesions in patients with RSSI using contrast-enhanced 3D HR-MRI. We found that atherosclerotic plaques were observed frequently at the MCA ipsilateral to the infarction lesions and were often located at the superior side of the vessel wall.

Assessment of lumen integrity is of limited value for the etiology of stroke because lumens are usually preserved by positive remodeling at the early stages of atherosclerosis. MRI of the vessel wall allows visualization of plaque morphology and characterization of plaque composition. Previous studies reported that positive arterial remodeling is more often associated with morphological predictors of plaque instability and plaque rupture [18, 19]. In our study, all 18 patients showed no obvious stenosis on time-of-flight MRA. However, with our 3D HR-MRI vessel wall imaging technique, we found 21 plaques in the patients, suggesting the presence of remodeling in the MCAs.

In patients with RSSI, plaques were present in 72.2% of the ipsilateral MCAs. This percentage of plaque is higher than that reported in previous studies (45.6–50%) [6, 7]. Such a high prevalence may be due to the use of novel 3D isotropic high-resolution vessel wall imaging. The 3D image sets allow multiplanar projection reconstruction (MPR) of images in arbitrary plane with no gap or image blurring. Compared with conventional 2D techniques [9], the ability to visualize vessel walls in various orientations improves the detection of plaques. Another advantage of 3D vessel wall imaging is that it further improves spatial coverage from the ostium of the MCA to the far end of the distal part [20]. In this regard, we found three cases in which plaques were located at the bifurcation of the M1 segment of the MCA, ipsilateral to the RSSI, which would have been missed by conventional 2D techniques. These findings are meaningful since lateral lenticulostriate arteries can originate at the bifurcation of the MCA or the nearby regions [21]. Our results therefore suggest that large artery disease may be an important cause of RSSI. The RSSI with relevant plaques in our study would be regarded as small artery diseases using other classification methods such as TOAST (Trial of ORG 10172 in Acute Stroke Treatment) [22] and SSS-TOAST (Stop Stroke Study-TOAST) [23] using conventional imaging techniques. However, these cases would have been classified as large artery disease according to the Chinese ischemic stroke subclassification [24]. Thus, 3D high-resolution vessel wall imaging would be a useful tool for etiologic assessment of ischemic stroke.

3D HR-MR can cover the entire range of bilateral MCAs in less than ten minutes and is more time-efficient compared with conventional 2D techniques. It allows the comparison between the ipsilateral MCAs of symptomatic RSSI with the contralateral MCAs. We found that plaques on both sides of RSSI were mostly superiorly located (92.3% on the ipsilateral side versus 83.3% on the contralateral side). As plaques occurred more often on the ipsilateral (72.2%) than on the contralateral sides (33.3%), the percentage of superiorly located plaques on the MCA ipsilateral to the RSSI was higher than that contralateral to the RSSI (66.7% versus 27.8%; *P* = 0.044). The superiorly located plaques commonly involve the orifices of penetrating arteries and may play an important role in the development of RSSIs [25]. A previous study reported that SSIs may be caused by perforator occlusion due to an atheroma at the MCA [26]. In light of these findings, our results support the association between superiorly located plaques and the RSSI.

**Figure 3:**
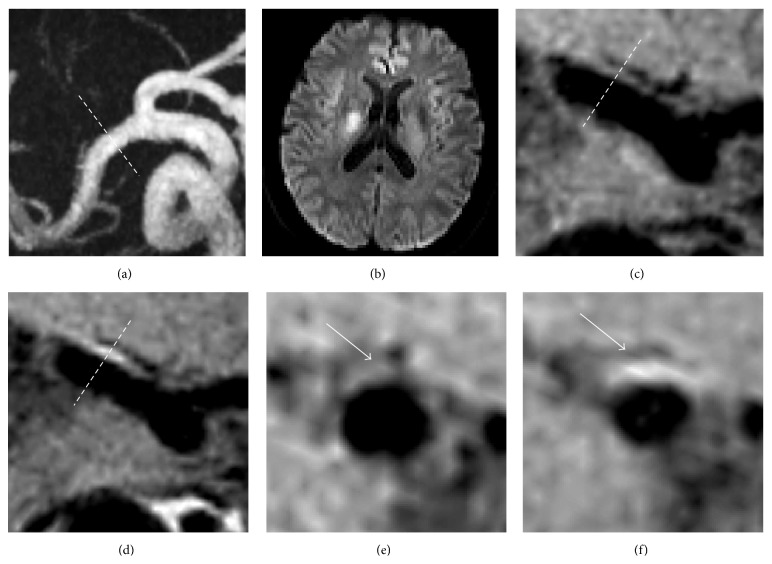
A 59-year-old man with left-side weakness. (a) MRA shows no stenosis on right MCA; (b) the diffusion-weighted image shows a hypertensive lesion in the right lenticular nucleus which extends to the coronal radiate; precontrast T1-weighted SPACE images did not show eccentric wall thickening clearly on both the long axis (c) and short axis ((e), arrow) of the MCA; postcontrast T1-weighted SPACE images show wall thickening ipsilateral to the infarct on both the long axis (d) and short axis ((f), arrow) of the MCA.

Contrast enhancement of the plaque has been recognized as an important marker of vulnerability in extracranial carotid and coronary arteries [27, 28]. A previous study showed that coronary wall enhancement in patients with acute myocardial infarction was associated with elevated systemic inflammatory markers [28]. The use of contrast agent in our study for intracranial vessel wall imaging helps the detection of the probable culprit plaques in stroke patients. We found that the enhancement patterns were consistent and largely confined to the vessels supplying the area of acute infarction (Figure 3). Unexpectedly, the mean percentage increases in SI of superiorly located plaques on the ipsilateral and contralateral sides of RSSI were very similar (58.8% versus 72.2%). Several recent small sample size studies found that enhancement of plaques was strongly associated with ischemic events [29–31]. However, Klein et al. had reservations about such associations [32]. Compared to the plaques in previous contrast enhancement studies where patients have atherosclerotic plaques leading to stenosis of intracranial arteries, the plaques in our study were small and nonstenotic. Note that the percentage of enhanced plaques also depended on the assessment methods, which may be done qualitatively (comparison with pituitary [29]) or quantitatively [30]. Here, the quantitative method used to assess plaque enhancement together with the small number of contralateral MCA plaques might lead to bias on the results relating plaque enhancement and RSSI. Whether the enhancement of MCA plaques is a good prognostic tool for ischemic stroke needs to be confirmed in a larger patient cohort and follow-up studies. Nevertheless, contrast-enhanced 3D HR-MRI has the potential to help subclassify stroke more accurately and unravel the pathogenic mechanism of SSI. Such new information may have a significant impact on the treatment strategy for patients. For instance, aggressive treatment to optimize plaque stabilization, including intensive lipid-lowering and anti-inflammatory medications, may be more appropriate for patients with superiorly located enhanced plaques.

There were several limitations in our study. First, the sample size was relatively small and data from age-matched healthy control subjects were unavailable for comparison. Second, the plaques that were detected cannot be verified by histopathology. Also, the culprit plaques could not be identified as the branch artery ostia cannot be clearly visualized in the images. Third, the spatial resolution of the images varied among different patients (isotropic voxel size varied from 0.125 to 0.343 mm^3^). Despite the varying voxel size used in different patients, the lowest spatial resolution in terms of voxel size in this study is still comparable to other studies using 2D techniques where the voxel sizes were in the range of 0.33 mm^3^ to 0.36 mm^3^. Additionally, our study population included only symptomatic patients with a normal lumen, and the results may not be directly applicable to patients with MCA stenosis caused by atherosclerotic plaques.

## 5. Conclusions

The current findings indicate that 3D high-resolution vessel wall imaging improves the visualization of MCA plaques and holds promise as a valuable alternative to current 2D MRI techniques. Our results suggest that atherosclerosis may be more prevalent among patients with RSSI than commonly believed. Superiorly located MCA plaques are associated with ipsilateral infarctions and may have an important role in the pathogenesis of RSSI. Plaques found in these patients with RSSI are mostly enhanced. Further studies are required to explore the relevance of these findings to the subclassification of stroke.

## Figures and Tables

**Table 1:** Demographic and baseline characteristics of the patients.

	RSSI (*n* = 18)
Age, median (range)	54.6 (40–70)
Male gender, %	16 (88.9)
Hypertension, %	12 (66.7%)
Diabetes, %	4 (22.2%)
Hyperlipidemia, %	10 (55.6%)
Current smoker, %	13 (72.2%)
NIHSS, median (range)	2.78 (0–12)
Maximum infarction diameter, mm, median (range)	14.7 (8.8–20)
Time from stroke onset to the second MRI, days, median (range)	12 (3–28)

Except for ranges, values are counts (percentages).

**Table 2:** Presence and positions of atherosclerotic plaques around the ipsilateral and contralateral MCAs.

Patient number	Infarction site	Ipsilateral				Contralateral			
		Precontrast		Postcontrast		Precontrast		Postcontrast	
		Presence	Position	Presence	Position	Presence	Position	Presence	Position
1	Left	Yes	Down	Yes	Up + down	No		No	
2	Left	Yes	Up	Yes	Up	Yes	Up	Yes	Up
3	Left	No		No		No		No	
4	Left	Yes	Up	Yes	Up	Yes	Up	Yes	Up
5	Left	Yes	Up + down	Yes	Up	No		No	
6	Left	Yes	Down	Yes	Down	No		No	
7	Left	Yes	Up	Yes	Up	Yes	Up	Yes	Down
8	Left	Yes	Up	Yes	Up + down	No		No	
9	Left	No		No		No		No	
10	Left	Yes	Up + down	Yes	Up	No		No	
11	Left	Yes	Up	Yes	Up + down	No		No	
12	Right	No		No		No		No	
13	Right	No		No		No		No	
14	Right	Yes	Up + down	Yes	Up + down	Yes	Up + down	Yes	Up + down
15	Right	No		Yes	Up	No		Yes	Up + down
16	Right	No		Yes	Up	No		Yes	Up + down
17	Right	No		No		No		No	
18	Right	No		Yes	Up	No		No	

**Table 3:** Ipsilateral and contralateral MCA wall findings.

Wall findings	Ipsilateral MCA	Contralateral MCA	*P* value
	(*n* = 18)	(*n* = 18)	
Presence of plaque	13 (72.2%)	6 (33.3%)	**0.044**
Superior wall	12 (66.7%)^*∗*^	5 (27.8%)^*∗∗*^	**0.044**
Inferior wall	5 (27.8%)	4 (22.2%)	1.000

Values are counts (percentages). Statistically significant values are shown in bold font.

MCA: middle cerebral artery.

^*∗*^Of the 12 superiorly located plaques, 4 are also inferiorly involved.

^*∗∗*^Of the 5 superiorly located plaques, 3 are also inferiorly involved.

**Table 4:** Enhancement of superiorly located plaques between ipsilateral and contralateral MCA.

Enhancement	Ipsilateral MCA	Contralateral MCA
	(*n* = 12)	(*n* = 5)
Absence (<20%)	1 (8.3%)	1 (20.0%)
Presence (≥20%)	11 (91.7%)	4 (80.0%)

Values are counts (percentages).
